# Predictors of mental health difficulties and subjective wellbeing in adolescents: A longitudinal study

**DOI:** 10.1002/jcv2.12074

**Published:** 2022-05-18

**Authors:** Suzet Tanya Lereya, Praveetha Patalay, Jessica Deighton

**Affiliations:** ^1^ Evidence‐Based Practice Unit University College London and Anna Freud National Centre for Children and Families London UK; ^2^ Centre for Longitudinal Studies and MRC Unit for Lifelong Health and Ageing University College London London UK

**Keywords:** determinants, longitudinal, mental health, psychiatric epidemiology, psychopathology, wellbeing

## Abstract

**Background:**

Mental health and subjective well‐being are of great interest in both health policy and research. There has been considerable debate regarding whether mental health difficulties and subjective wellbeing are two distinct domains or different ends of a single mental health spectrum. This study investigates if predictors of mental health difficulties and subjective wellbeing are the same or different in a large‐scale community‐based sample in the United Kingdom.

**Methods:**

13,500 adolescents in year 7 (aged 11–12) and again in year 8 (aged 12–13) completed surveys on emotional strengths and skills, support networks, mental health difficulties and wellbeing. Socio‐demographic factors were gathered from the National Pupil Database. Mental health difficulties and wellbeing scores were standardized to allow comparisons.

**Results:**

The correlation between mental health difficulties and subjective wellbeing was −0.48, indicating a moderate overlap between the two domains. Some of the predictors (e.g., gender, ethnicity, problem solving, emotion regulation) in year 7 predicted both mental health difficulties and subjective wellbeing in year 8. However, some of the predictors in year 7 only predicted mental health difficulties (e.g., special education needs, empathy) and some only subjective wellbeing (e.g., prosocial behaviour, peer support) in year 8.

**Conclusion:**

This study provides further evidence for differences in what predicts adolescents’ mental health difficulties and subjective wellbeing. It highlights the importance of not only focusing on preventing or treating symptoms of mental illness but also focusing on improving children’s wellbeing.

## Graphical abstract

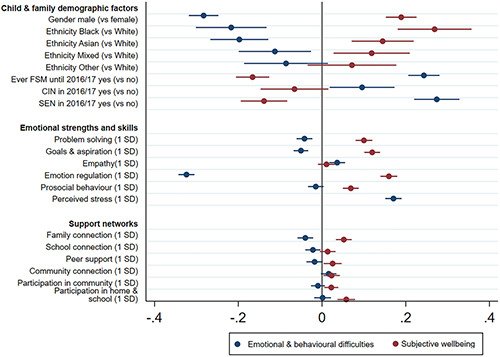

This study investigated if predictors of mental health difficulties and subjective wellbeing are the same or different in a large‐scale community‐based sample in the United Kingdom. The results showed that some of the predictors (e.g., gender, ethnicity, emotion regulation) in year 7 predicted both mental health difficulties and subjective wellbeing in year 8. Some of the predictors in year 7 only predicted mental health difficulties (e.g., special education needs, empathy) and some only predicted subjective wellbeing (e.g., prosocial behaviour, peer support) in year 8.

**Key points**

- By using different reporters for mental health difficulties and subjective wellbeing, previous research has identified overlapping and diverse correlates of mental health difficulties and subjective wellbeing. This research extends the previous research by focusing on predictors of mental health difficulties and subjective wellbeing using children's self‐reports
- The results showed a moderate association between mental health difficulties and subjective wellbeing at year 8 (aged 12–13)
- Only certain predictors (i.e., gender, being Asian or Black (compared to being White), free school meal eligibility, problem solving, goals & aspiration, emotion regulation, perceived stress and family connection) predicted both mental health difficulties and subjective wellbeing
- It is important not to only focus on preventing or treating symptoms of mental illness but also focusing on improving children's wellbeing. Especially, interventions designed to improve mental health should also consider having elements to improve wellbeing

## INTRODUCTION

Adolescence is a critical period for young people's mental health and wellbeing (Dahl et al., 2018; Patton et al., 2016). The majority of mental health disorders show first onset before the age of 25 (Jones, 2013). Furthermore, adolescents perceive lower levels of life satisfaction and experience emotional distress more frequently and with greater intensity than younger children or older adults (Arnett, 2008). Understanding the predictors of mental health disorders in childhood can provide insight into what sets changes in mental health in motion. Recent research estimates that one in eight children and young people experience mental health problems in the UK (NHS Digital, 2020). Mental health disorders experienced in adolescence can cause a high burden for both the society and the individuals and can lead to significant impairments in various areas of life such as family life and professional life as well as quality of life (Copeland et al., 2015).

There are two main distinct yet overlapping perspectives on wellbeing: one focuses on hedonic wellbeing and the other on eudaimonic wellbeing (Lambert et al., 2015; Ryan & Deci, 2001). According to the hedonic philosophical tradition, subjective wellbeing is a person's evaluation and feelings of their own life (Diener, 1984) and refers to the levels of positive affect, low levels of negative affect, and a high degree of overall life satisfaction (Diener & Lucas, 1999). On the other hand, the eudaimonic perspective refers to wellbeing as distinct from happiness and states that it occurs when individuals' life activities are congruent with deeply held values, which are holistically or fully engaged (Waterman, 1993). Some regard wellbeing as requiring both hedonic and eudaimonic components; that is, the combination of feeling good and functioning well (Huppert & So, 2013). For example, Seligman (2011) proposed the PERMA model to describe wellbeing, which is the acronym for positive emotion, engagement, relationships, meaning, and accomplishment.

Some consider wellbeing and mental ill‐health to be two ends of the same spectrum of mental health. However, World Health Organization defined health as “a state of complete physical, mental and social wellbeing and not merely the absence of disease or infirmity” (World Health Organization, 1948, p. 1). Moreover, there has been identification of individuals who experience good wellbeing in the presence of symptoms of mental ill‐health (Kinderman et al., 2015; Sharpe et al., 2016) and many individuals otherwise free of mental disorder do not feel healthy or function well (Keyes, 2005). The Complete State Model of Mental Health (Keyes & Lopez, 2002) combines the two spectrums. It states that an individual may have high mental illness symptoms and low wellbeing “floundering” which is defined as complete mental illness, or they may have high mental illness symptoms but high wellbeing “struggling” which is defined as incomplete mental illness. On the other hand, they may have low mental illness symptoms and low wellbeing “languishing” which is described as incomplete mental health, or they may have low mental illness symptoms and high wellbeing “flourishing” which is defined as complete mental health. In line with diagnostic criteria for a mental disorder not requiring all the symptoms to be present, the operational definitions of flourishing (Huppert & So, 2013; Keyes, 2002) may not require all the features of positive feeling and functioning to be present which would allow for an overlap between symptoms of mental disorder and features of flourishing (Huppert, 2014).

Depending on the theoretical framework, each researcher prioritises different components and develops a measurement scale (Huppert, 2014). However, there are also measures that have been developed by reviewing existing scales and items assessing wellbeing and related constructs such as the widely used Warwick–Edinburgh Mental Wellbeing Scale (WEMWBS; Stewart‐Brown et al., 2009). The WEMWBS focuses on the main components of mental wellbeing, defined as ‘feeling good and functioning well’. It includes both hedonic and eudaimonic perspectives on wellbeing. On the other hand, the Short Warwick–Edinburgh Mental Well‐being Scale (SWEMWBS) focuses more on the psychological functioning rather than the subjective feeling states (Ng Fat et al., 2017).

The relationship between mental health difficulties and subjective wellbeing has been investigated in adult populations. Twin studies have shown evidence of some shared variance between mental illness and wellbeing. Kendler et al. (2011) have shown that for internalising psychopathology (prevalence of clinical levels of major depression, generalized anxiety or panic disorder in the previous year), genetics played a larger role than environment in the shared associations between wellbeing and internalising psychopathology. Moreover, Kendler et al. (2011) found that the strongest risk factors for low mental wellbeing were genetic factors that impact on both internalising psychopathology and externalising psychopathology and environmental factors that impact on externalising psychopathology (defined as alcohol‐related problems and smoking). Similarly, Bartels et al. (2013) showed that the overlap between psychopathology and wellbeing was mainly genetic. Hofgaard et al. (2021) investigated the contributors to illbeing and wellbeing within a twin study design. They have identified that the genetic correlation between promotion of wellbeing and prevention of illbeing, despite stress, was 0.43 and the heritability was 0.24 and 0.30 respectively. Different factors were common to both prevention of illbeing and promotion of wellbeing (finding meaning in daily activities, a perception of good health, experiencing positive affect, being optimistic, not feeling lonely, and believing in one's capacity to handle difficult demands), while others were found to represent unique effects on either prevention of illbeing (being physically active and trusting others) or promotion of wellbeing (feeling satisfied and secure in close relationships). Studies that investigate the association between mental health difficulties and wellbeing are fewer across the adolescent populations. A study by Patalay and Fitzsimons (2016), investigated a wide range of individual, family, social and wider environmental predictors of mental health difficulties and wellbeing for 11‐year‐olds using a large, nationally representative sample (Millennium Cohort Study, MCS). Their results showed that alongside some factors that predicted both outcomes (e.g., sex, being from a single parent family, arguing with parents, sibling bullying and peer problems), many factors were uniquely associated with either children's mental illness (e.g., ethnicity, cognitive ability, special education needs, communication difficulties and chronic illness) or subjective wellbeing (e.g., being overweight, parent problems, being bullied by peers and having a safe neighbourhood). While this study provides further evidence for mental health problems and wellbeing as distinct constructs, some methodological shortcomings limit conclusions. Notably, although subjective wellbeing was reported by the adolescents themselves, mental health difficulties were parent reported. Hence, the uniquely associated factors for mental health difficulties and wellbeing might have been due to different reporter perspectives. A further study by Rees (2018) investigated the predictiveness of early childhood (age 5) family and socio‐economic factors on children's subjective wellbeing and emotional and behavioural difficulties at the age of 11. Similar to the study by Patalay and Fitzsimons (2016), their results showed predictors that are uniquely associated with subjective wellbeing and emotional and behavioural difficulties. However, this study also relied on the MCS and, therefore, shared the same limitation of different reporters as the Patalay and Fitzsimons' study (Patalay & Fitzsimons, 2016).

Current study draws on a recent, large‐scale community‐based self‐report survey of adolescents to explore the correlates of mental health difficulties (emotional and behavioural difficulties) and subjective wellbeing. Specifically, child and family demographic factors, child reported emotional strengths and skills and support networks in year 7 were used to predict child‐reported mental health difficulties and subjective wellbeing in Year 8.

## METHODS

### Participants

This study investigated data that were collected between 2016/17–2017/18 from children who were part of a programme called HeadStart. Started in 2016, HeadStart is a 6‐year, £67.4 million National Lottery funded programme set up by The National Lottery Community Fund. HeadStart aims to explore and test new ways to improve the mental health and wellbeing of young people aged 10 to 16 and prevent serious mental health issues from developing. To do this, six local authority led HeadStart partnerships (Blackpool, Cornwall, Hull, Kent, Newham and Wolverhampton) are working with local young people, schools, families, charities, community and public services to design and try out new interventions aiming to promote young people's mental health, wellbeing, and resilience. Schools have been selected by these partnerships; in most cases driven by perceived need in these schools but also by the potential for schools to successfully engage with the programme. The analyses reported are based on 13,500 students who completed annual surveys (The Wellbeing Measurement Framework, WMF) in year 7 (aged 11–12) and again in year 8 (aged 12–13). The socio‐demographic characteristics of children were extracted through a data linkage with the National Pupil Database (NPD). The sample was not drawn to be representative of all school children in England; it was based on local areas that were part of the HeadStart programme (Big Lottery Fund, 2016). Compared to the national average, the study sample had a slightly higher proportion of children/young people from deprived backgrounds based on free school meal (FSM) eligibility (study sample: 16.04%, national average: 12.9%). The study sample had much lower proportion of children with special education need (SEN) support (study sample: 11.59%, national average: 14.4%), slightly higher proportion of White children/young people (study sample: 77.3%, national average: 75.2%) and females (study sample 53.6%, national average: 49.3%).

### Measures

#### Main outcomes

At year 8 (aged 12–13), mental health difficulties was measured with the aggregated Emotional Symptoms and Conduct Problems subscales of the Strengths and Difficulties Questionnaire (SDQ; Goodman et al., 1998) which is a widely used questionnaire of psychopathology symptoms in the United Kingdom since the late 1990s. Higher scores indicate greater symptoms.

Subjective wellbeing was measured with the 7‐item child self‐report Short Warwick and Edinburgh Wellbeing Scale (SWEMWBS) when the adolescents were in year 8 (aged 12–13; Stewart‐Brown et al., 2009). High scores on the SWEMWBS indicate greater positive subjective wellbeing.

#### Predictors

Three key areas were investigated: 1) child and family sociodemographic factors; 2) emotional strengths and skills (measured in year 7); and 3) support networks (measured in year 7). The variables included in each area are detailed below. Descriptive statistics (proportions for categorical variables and means for continuous scale scores) are presented in Table 1.

**TABLE 1:** Descriptive statistics and missing data for all study variables

Block	Predictors	% With missing data (out of *n* = 13,500)	Descriptive statistics original sample% or mean (95% CI)	Descriptive statistics imputed sample% or mean (95% CI)
Outcomes	Emotional & behavioural difficulties	1.69	6.41 (6.34, 6.47)	6.42 (6.36, 6.49)
	Subjective wellbeing	9.76	22.12 (22.04, 22.20)	22.08 (22.00, 22.16)
Child and family demographic factors	Gender (female)^a^	0	53.60	–
	Ethnicity	3.01	–	–
	White^a^	–	77.31	77.29 (76.57, 78.01)
	Asian	–	10.05	10.08 (9.56, 10.60)
	Black	–	5.61	5.59 (5.21, 5.99)
	Mixed	–	4.02	4.02 (3.68, 4.35)
	Other	–	3.01	3.01 (2.72, 3.30)
	Free school meal (FSM) eligibility until 2016/17 (no)^a^	3.01	65.17	65.15 (64.33, 65.96)
	Child in need (CIN) status in 2016/17 (no)^a^	0	95.00	–
	Special education need (SEN) eligibility in 2016/17 (no)^a^	4.13	88.28	88.80 (87.47, 88.60)
Emotional strengths and skills	Problem solving	6.62	11.10 (11.04, 11.16)	11.07 (11.02, 11.13)
	Goals & aspiration	6.24	8.29 (8.25, 8.32)	8.27 (8.24, 8.31)
	SRS empathy	5.81	8.27 (8.24, 8.30)	8.25 (8.22, 8.29)
	Emotion regulation	8.93	26.10 (25.96, 26.23)	26.04 (25.91, 26.17)
	Prosocial behaviour	1.95	7.56 (7.53, 7.59)	7.55 (7.52, 7.59)
	Perceived stress	8.70	6.47 (6.41, 6.52)	6.48 (6.42, 6.53)
Support networks	Family connection	4.96	17.88 (17.83, 17.92)	17.84 (17.80, 17.89)
	School connection	5.69	15.38 (15.31, 15.44)	15.37 (15.31, 15.43)
	Peer support	9.30	52.89 (52.69, 53.09)	52.67 (52.47, 52.86)
	Community connection	5.54	17.39 (17.33, 11.44)	17.36 (17.30, 17.42)
	Participation in community life	6.47	7.28 (7.23, 7.33)	7.26 (7.21, 7.30)
	Participation in home and school life	7.21	13.61 (13.55, 13.67)	13.59 (13.53, 13.65)

*Note*: Descriptive statistic is the mean but standardised scores are used in the analysis for all the continuous variables.

^a^ Reference group in the analysis.

#### Child and family sociodemographic factors

The child and family socio‐demographic factors were extracted through a data linkage with the NPD. Child demographic characteristics included sex (coded 0 = female, 1 = male), ethnicity grouped into 5 broad groupings (White, Asian, Black, mixed, and other ethnic groups), child in need status (CIN; coded 0 = no, 1 = yes) in 2016/17 referring to children's social care, most frequently because of concerns about abuse or neglect, acute family stress or familial dysfunction. Free school meals until 2016/17 (FSM; coded 0 = no, 1 = yes) which is frequently used as an indicator of low family income, since only families on income support are eligible, and SEN eligibility in 2016/17 (coded 0 = no, 1 = yes) were extracted from NPD data linkage.

#### Emotional strengths and skills

Problem solving skills, goals & aspirations, and empathy were measured using the Student Resilience Survey (SRS; Lereya et al., 2016). Higher scores on the SRS reflect better outcomes. Prosocial behaviour has been measured with prosocial subscale of the SDQ (R. Goodman et al., 1998), higher scores indicate greater prosocial behaviour. Emotion regulation has been measured with the Trait Emotional Intelligence Questionnaire‐Adolescent Short Form (TEIQUE‐ASF; Petrides & Furnham, 2009), and perceived stress has been measured with the 4‐item perceived stress scale (PSS; Demkowicz et al., 2020). Higher scores on the TEIQUE‐ASF reflect greater emotion regulation and higher scores on the PSS indicates higher stress.

#### Support networks

Family connection, school connection, peer support, community connection, participation in community life and participation in home and school life were measured using the SRS (Lereya et al., 2016). Higher scores on the SRS reflect better outcomes.

### Procedure

Every year, children in participating schools completed surveys using a secure online system during a usual school day. The online system was designed to be easy to read and child friendly. Ethical approval was granted by the UCL ethics committee (reference: 8097/003) and every year, consent for participation in the research was sought from parents prior to, and from children and young people at the outset of, the survey sessions. Parental opt‐outs were received by post, phone or email and child assent was recorded via computer at the beginning of survey sessions.

### Statistical analysis

Multiple imputation with chained equations (*n* = 20) were used to impute missing values (using the mi package in Stata 15). To ensure plausibility of the missing at random assumption, our imputation model included all the predictors and outcomes across the 3 years of HeadStart data collection (2016/17 to 2018/19). Overall, missing cells were at 34.7% of the total, with missingness varying from 1.7 for mental illness to 9·8% for wellbeing (for more information see Table 1). To investigate whether the predictors of mental health difficulties and subjective wellbeing are the same or different, regression analyses were conducted predicting standardized scores (z‐scores) for mental health difficulties and wellbeing allowing for comparison of coefficient sizes across the 2 models (Table 2). Random effects linear regression analyses (allowing for different school intercepts) were conducted and for each of the study outcomes. First model (Model A) examined the child and family demographic factors including gender, ethnicity, FSM, CIN, and SEN. Second model (Model B) factors related to emotional strengths and skills (i.e., problem solving, goals & aspiration, empathy, emotion regulation, prosocial behaviour and perceived stress) were included and finally in the last model (Model C) factors related to support networks (i.e., family connection, school connection, peer support, community connection, participation in community life and participation in home and school life) were included.

**TABLE 2:** Coefficients (95% CI) from regression models predicting symptoms of mental health difficulties and wellbeing

		Mental health difficulties						Subjective wellbeing					
		Model A		Model B		Model C		Model A		Model B		Model C	
Block	Predictors	Estimate (95% CI)	P	Estimate (95% CI)	P	Estimate (95% CI)	P	Estimate (95% CI)	P	Estimate (95% CI)	P	Estimate (95% CI)	P
Child and family demographic factors	Gender (male)^a^	**−0.28 (−0.32, −0.25)**	**<0.0001**	**−0.25 (−0.28, −0.22)**	**<0.0001**	**−0.26 (−0.29, −0.22)**	**<0.0001**	**0.19 (0.15, 0.23)**	**<0.0001**	**0.20 (0.16, 0.24)**	**<0.0001**	**0.21 (0.17, 0.24)**	**<0.0001**
	Ethnicity (Asian)^b^	**−0.20 (−0.27, −0.13)**	**<0.0001**	**−0.12 (−0.18, −0.06)**	**<0.0001**	**−0.13 (−0.19, −0.07)**	**<0.0001**	**0.14 (0.07, 0.22)**	**<0.0001**	**0.09 (0.03, 0.16)**	**0.005**	**0.11 (0.04, 0.17)**	**0.001**
	Ethnicity (Black)^b^	**−0.22 (−0.30, −0.13)**	**<0.0001**	**−0.15 (−0.22, −0.07)**	**<0.0001**	**−0.15 (−0.22, −0.07)**	**<0.0001**	**0.27 (0.18, 0.36)**	**<0.0001**	**0.22 (0.15, 0.31)**	**<0.0001**	**0.22 (0.14, 0.29)**	**<0.0001**
	Ethnicity (Mixed)^b^	**−0.11 (−0.20, −0.03)**	**0.011**	−0.06 (−0.13, 0.02)	0.127	−0.06 (−0.13, 0.02)	0.135	**0.12 (0.03, 0.21)**	**0.011**	**0.09 (0.00, 0.17)**	**0.041**	0.08 (−0.00, 0.16)	0.058
	Ethnicity (Other)^b^	−0.09 (−0.19, 0.01)	0.093	−0.07 (−0.16, 0.02)	0.130	−0.07 (−0.15, 0.02)	0.129	0.07 (−0.03, 0.18)	0.186	0.05 (−0.05, 0.14)	0.342	0.04 (−0.05, 0.14)	0.353
	Ever FSM until 2016/17 (yes)^c^	**0.24 (0.21, 0.28)**	**<0.0001**	**0.14 (0.11, 0.18)**	**<0.0001**	**0.14 (0.11, 0.17)**	**<0.0001**	**−0.17 (−0.21, −0.13)**	**<0.0001**	**−0.07 (−0.10, −0.04)**	**<0.0001**	**−0.06 (−0.10, −0.03)**	**0.001**
	CIN in 2016/17 (yes)^e^	**0.10 (0.02, 0.17)**	**0.015**	0.04 (−0.02, 0.11)	0.211	0.04 (−0.00, 0.14)	0.232	−0.07 (−0.15, −0.02)	0.112	−0.02 (−0.10, 0.05)	0.606	−0.01 (−0.10, 0.06)	0.741
	SEN in 2016/17 (yes)^d^	**0.27 (0.22, 0.33)**	**<0.0001**	**0.12 (0.07, 0.17)**	**<0.0001**	**0.12 (0.07, 0.16)**	**<0.0001**	**−0.14 (−0.19, −0.08)**	**<0.0001**	−0.00 (−0.05, 0.05)	0.975	0.01 (−0.04, 0.06)	0.637
Emotional strengths and skills	Problem solving			**−0.04 (−0.06, −0.02)**	**<0.0001**	**−0.03 (−0.05, −0.01)**	**0.006**			**0.10 (0.08, 0.12)**	**<0.0001**	**0.06 (0.04, 0.08)**	**<0.0001**
	Goals & aspiration			**−0.05 (−0.07, −0.03)**	**<0.0001**	**−0.04 (−0.06, −0.02)**	**<0.0001**			**0.12 (0.10, 0.14)**	**<0.0001**	**0.09 (0.07, 0.10)**	**<0.0001**
	Empathy			**0.04 (0.02, 0.05)**	**<0.0001**	**0.04 (0.02, 0.06)**	**<0.0001**			0.01 (−0.01, 0.03)	0.307	−0.01 (−0.03, 0.01)	0.225
	Emotion regulation			**−0.32 (−0.34, −0.30)**	**<0.0001**	**−0.32 (−0.34, −0.30)**	**<0.0001**			**0.16 (0.14, 0.18)**	**<0.0001**	**0.15 (0.13, 0.17)**	**<0.0001**
	Prosocial behaviour			−0.01 (−0.03, 0.00)	0.115	−0.01 (−0.03, 0.01)	0.481			**0.07 (0.05, 0.09)**	**<0.0001**	**0.05 (0.03, 0.07)**	**<0.0001**
	Perceived stress			**0.17 (0.15, 0.19)**	**<0.0001**	**0.16 (0.14, 0.18)**	**<0.0001**			**−0.18 (−0.20, −0.16)**	**<0.0001**	**−0.17 (−0.19, −0.14)**	**<0.0001**
Support networks	Family connection					**−0.04 (−0.06, −0.02)**	**<0.0001**					**0.05 (0.03, 0.07)**	**<0.0001**
	School connection					**−0.02 (−0.04, −0.00)**	**0.020**					0.01 (**−**0.01, 0.03)	0.159
	Peer support					**−**0.02 (**−**0.04, 0.00)	0.073					**0.03 (0.00, 0.05)**	**0.022**
	Community connection					0.02 (**−**0.00, 0.03)	0.095					**0.02 (0.00, 0.04)**	**0.024**
	Participation in community life					**−**0.01 (**−**0.03, 0.01)	0.250					**0.02 (0.01, 0.04)**	**0.010**
	Participation in home and school life					0.00 (**−**0.02, 0.02)	0.890					**0.06 (0.04, 0.08)**	**<0.0001**

*Note*: Each column represents a new regression model. All models were tested using multilevel linear regression. The bold values are to show significant findings.

^a^ Reference category is male.

^b^ Reference category is White.

^c^ Reference category is those without eligibility to free school meals.

^d^ Reference category is those without special education need status.

^e^ Reference category is those without child in need status.

## RESULTS

Table 1 presents descriptive statistics for all the study variables. The majority of the correlations between the predictor variables were below ±0.49. The exceptions were school connection (which correlated 0.50 with peer support and 0.54 with participation in home and school life), and prosocial behaviour (which correlated 0.55 with empathy) and perceived stress (which correlated −0.59 with emotion regulation). The correlation between the two outcome variables, mental health difficulties and subjective wellbeing, was −0.48, indicating a moderate overlap between the two domains. The variables included in the model predicted 27% of the variance in mental difficulty and 24% of the variance in subjective wellbeing.

**FIGURE 1:**
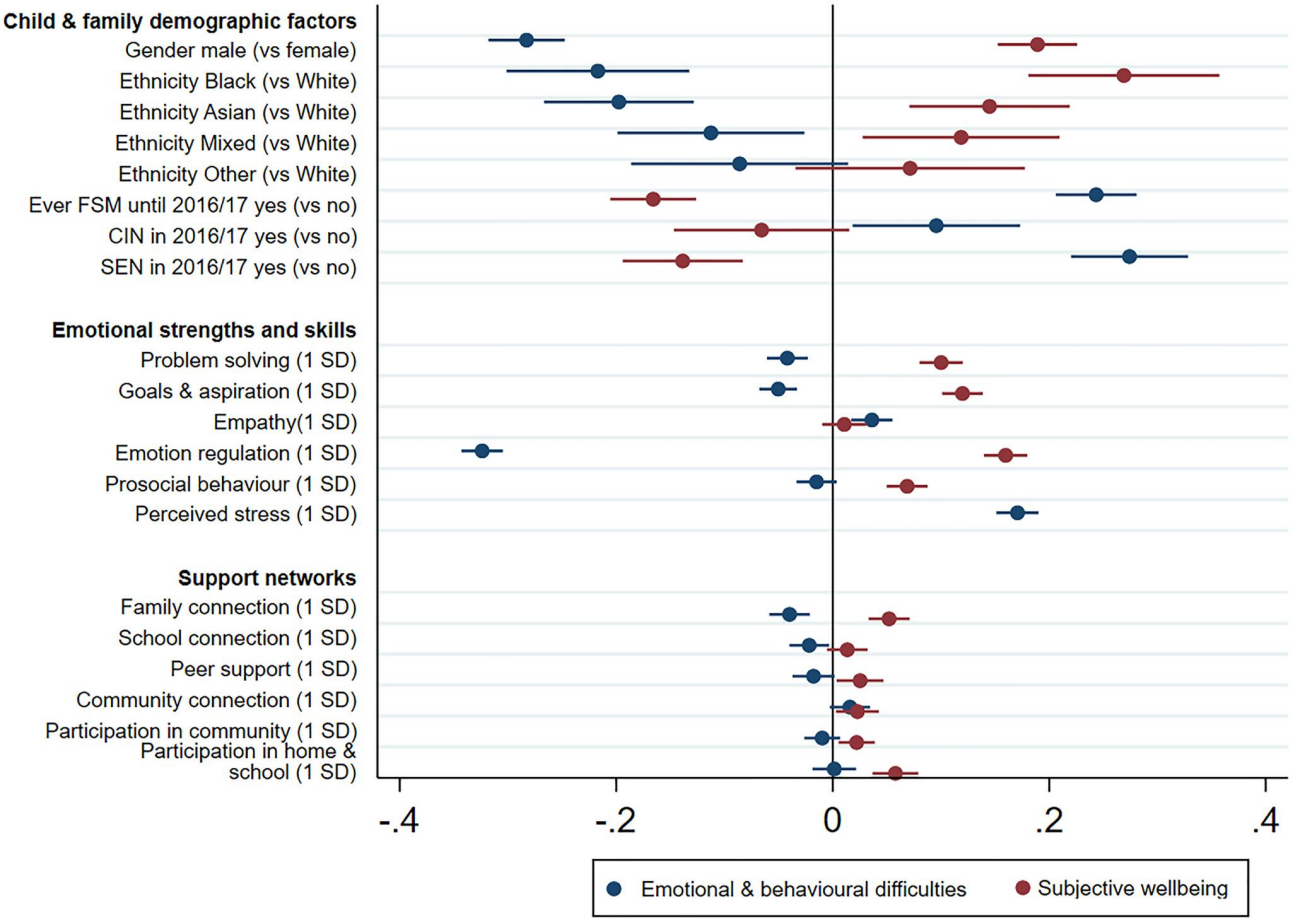
Regression coefficients predicting mental illness and subjective wellbeing

Table 2 presents regression results for mental health difficulties and wellbeing. From the predictors, special education needs (*β* = 0.12; CI = 0.07 to 0.16), empathy (*β* = 0.04; CI = 0.02 to 0.06) and school connection (*β* = −0.02 CI = −0.04 to −0.00) at year 7 uniquely predicted mental health difficulties in year 8. Prosocial behaviour (*β* = 0.05; CI = 0.03 to 0.07), peer support (*β* = 0.03; CI = 0.00 to 0.05), community connection (*β* = 0.02; CI = 0.00 to 0.04), participation in community life (*β* = 0.02; CI = 0.01 to 0.04) and participation in home and school life (*β* = 0.06; CI = 0.04 to 0.08) in year 7 uniquely predicted subjective wellbeing in year 8. Gender, being Asian or Black (compared to being White), FSM, problem solving, goals & aspiration, emotion regulation, perceived stress and family connection in year 7 predicted both mental health difficulties and subjective wellbeing in year 8 (see Table 2 for the regression coefficients). Figure 1 displays the regression coefficients from respective regressions when the model is first added in to predict standardized scores of mental illness and subjective wellbeing.

## DISCUSSION

There is a growing interest in the study of subjective wellbeing of children and adolescents (Holder, 2012; Patalay & Fitzsimons, 2016, 2018; Rees, 2018). The current study investigated which child and family demographic factors, emotional strengths and skills, and support networks measured in year 7 predicted adolescents mental health difficulties and subjective wellbeing in year 8.

The results showed that from the child and family demographic factors, being a boy was associated with lower mental health difficulties and higher subjective wellbeing across all the models. This is consistent with literature suggesting that girls are more likely to display depressive symptoms across most of the life span, beginning at some point in adolescence (Piccinelli & Wilkinson, 2000; Yoon et al., 2022) which might be due to earlier pubertal timing (Ge et al., 2001) and negative coping styles such as rumination (Nolen‐Hoeksema & Girgus, 1994). Being Asian or being Black, compared to being White, was associated with fewer mental health difficulties and better subjective wellbeing in the current study, which is in line with previous research (A. Goodman et al., 2008; Maynard & Harding, 2010). Consistent with research indicating that poverty is a potent predictor of both mental illness and poor wellbeing (Bradley & Corwyn, 2002), our results indicated that free school meal (FSM) eligibility, which is linked to low income, was associated with higher mental health difficulties and lower subjective wellbeing across all models.

Similar to previous findings, goals and aspirations (Deci & Ryan, 2000) and high problem‐solving skills were associated with low mental health difficulties and higher subjective wellbeing in the current analyses. Previous literature has suggested that problem solving skills is a major intrapersonal and interpersonal process that leads to quality relationships and an enhanced quality of life (Wallander et al., 2001). Similarly, and consistent with the findings presented here, emotion regulation has been linked with both mental health difficulties and subjective wellbeing (Kraiss et al., 2020). Indeed, trainings in social problem solving and emotion regulation are commonly used in remedial programs in schools and in the treatment of a wide variety of psychiatric disorders (Frey et al., 2000). Our results showed that perceived stress has been associated with both mental health difficulties and wellbeing. A previous systematic review has shown that stress is related to poorer quality of life by exacerbating various aspects of physical and mental health for university students (Ribeiro et al., 2017). On the other hand, the current analysis showed that high empathy was only associated with higher mental health difficulties and high prosocial behaviour was only associated with high subjective wellbeing. Previous research has shown that high levels of empathy may increase the vulnerability to mental health difficulties (O’Connor et al., 2007). With regards to prosocial behaviour, it has been long thought that prosocial behaviours affect the wellbeing of the helper as well as the help recipient. Indeed, previous research did identify positive effects of prosocial behaviour on the wellbeing of the helper (Weinstein & Ryan, 2010).

From the factors associated with support networks, only family connection was associated with both mental health difficulties and wellbeing in the current study. Previous research has shown that parent‐child relationship quality has been associated with mental health outcomes (Bukowski et al., 2006; Williams et al., 2009). Furthermore, according to the attachment theory, interactions within the family environment can enable or thwart the development of capabilities to build positive relationships elsewhere (Bowlby, 2005) which may lead to better mental health and wellbeing (Moore et al., 2018). Contrary to a previous finding (Patalay & Fitzsimons, 2016), and despite being moderately correlated to peer support and participation in home and school life, school connection was only related to mental health difficulties in the current analyses. The reason behind the difference may be due to the school connection measure used in this study being different than the one used in Patalay and Fitzsimons study. The findings from this study showed that having someone who cares about the individual, listens, and believes in them was associated with lower mental health difficulties. This is in line with the attachment theory suggesting that having a secure emotional connection to key individuals providing a base for psychological and social development (Berkman & Glass, 2000; Bowlby, 2005). Previous research also has shown that those with good school connectedness had the lowest risk of depressive symptoms (Bond et al., 2007). However, our findings indicated that high levels of peer support, community connection, participation in community life, and participation in home and school life only predicted high subjective wellbeing, not mental health problems.

There has been considerable debate regarding whether mental health difficulties and subjective wellbeing are two distinct domains or different ends of a single mental health spectrum. Previous research investigating the association between mental health difficulties and wellbeing showed a weak correlation (Patalay & Fitzsimons, 2016). However, it is important to note that although wellbeing was measured with children's self‐report whereas mental health difficulties were measured with parental report in this previous study. In the current study, both mental health difficulties and wellbeing were measured by adolescents' self‐report and the results showed a moderate correlation between the two domains (*r* = −0.48). This provides further evidence in support of the dual continuum model which views mental illness and wellbeing as two separate continua rather than opposite ends of the same continuum, and this evidence is not conflated by potential reporter difference effects (Keyes, 2002). Moreover, previous studies investigating the shared associations between wellbeing and mental health difficulties using twin study designs have shown that genetics play a significantly larger role than environment (Bartels et al., 2013), however there is limited evidence regarding the biological mechanisms and underpinnings of wellbeing and mental ill‐health and more research considering these constructs in varied age groups and diverse populations is needed. It is commonly assumed that classifying and monitoring mental health difficulties of adolescents is enough and adolescents without mental health difficulties are assumed to be homogenous, functioning about the same and markedly better than adolescents with mental health difficulties. The results from this study highlights the importance of also measuring wellbeing and promoting mental wellbeing alongside preventing and treating the symptoms of mental illness.

It is important to note the methodological limitations of the study. Firstly, the population of the study was not drawn to be representative of all school children in England; however, the participants were from six local areas of England which increased the generalisability of the results. Moreover, the associations and the coefficients are not expected to change in less disadvantaged groups. Secondly, even though self‐report is an acknowledged way of measuring adolescent mental health, social desirability may impact how children and young people complete the surveys and there is generally low to modest agreement between different reporters of child mental health problems (Cheng et al., 2018). Thirdly, while the longitudinal nature of the data made it possible to separate predictors from mental health and wellbeing, a causal relationship cannot entirely be inferred from these longitudinal associations. Lastly, no information about parent's marital status, parents' education level and number of siblings were available. Future research should include more family related factors. The key strength of the present study is the inclusion of both mental illness and wellbeing, which are reported by the same reporter (children themselves) using validated composite measures, SWEMWBS for subjective wellbeing and SDQ for mental health difficulties. Nevertheless, future studies should investigate the predictors of mental health difficulties and wellbeing via multi‐informants.

## CONCLUSIONS

In conclusion, the present study addresses the methodological limitations of previous studies and lends weight to the argument that mental health and wellbeing are related but distinct constructs with a number of distinct predictors. It identified factors that are distinctly related to mental health difficulties and subjective wellbeing and highlights the importance of not only focusing on preventing or treating symptoms of mental illness but also focusing on improving children's wellbeing. Future research should unpack the relationship between the factors identified in this study and consider interactions between predictors from different areas of life.

## CONFLICT OF INTEREST

The authors have declared that they have no competing or potential conflicts of interest.

## ETHICS STATEMENT

Ethical approval was granted UCL ethics committee (reference: 8097/003) and every year, consent for participation in the research was sought from parents prior to, and from children and young people at the outset of, the survey sessions. Parental opt‐outs were received by post, phone or email and child assent was recorded via computer at the beginning of survey sessions.

## AUTHOR CONTRIBUTIONS

Suzet Tanya Lereya; Conceptualization, analysis, Writing – original draft, Writing – review & editing; Praveetha Patalay; Conceptualization, Writing – review & editing; Jessica Deighton; Conceptualization, Supervision, Writing – review & editing.

## Data Availability

The data that support the findings of this study are available on request from the corresponding author. The data are not publicly available due to privacy or ethical restrictions.
